# Associating Patient Responses with Drug Sensitivity in Non-Small Cell Lung Carcinoma Using an Immunoassay on Patient-Derived Cell Cultures

**DOI:** 10.3390/cimb47040281

**Published:** 2025-04-17

**Authors:** Ana Podolski-Renić, Sofija Jovanović Stojanov, Dragana Marić, Jelena Dinić, Miodrag Dragoj, Ana Stepanović, Ema Lupšić, Milica Pajović, Sofija Glumac, Maja Ercegovac, Milica Pešić

**Affiliations:** 1 Department of Neurobiology, Institute for Biological Research “Siniša Stanković”—National Institute of the Republic of Serbia, University of Belgrade, Bulevar Despota Stefana 142, 11108 Belgrade, Serbia; ana.podolski@ibiss.bg.ac.rs (A.P.-R.); sofija.jovanovic@ibiss.bg.ac.rs (S.J.S.); jelena.dinic@ibiss.bg.ac.rs (J.D.); miodrag.dragoj@ibiss.bg.ac.rs (M.D.); ana.kostic@ibiss.bg.ac.rs (A.S.); ema.lupsic@ibiss.bg.ac.rs (E.L.); milica.pajovic@ibiss.bg.ac.rs (M.P.); 2 School of Medicine, University of Belgrade, Dr. Subotića 8, 11000 Belgrade, Serbia; dragana.maric@med.bg.ac.rs (D.M.); sofija.glumac@med.bg.ac.rs (S.G.); maja.ercegovac@med.bg.ac.rs (M.E.); 3 Clinic for Pulmonology, University Clinical Center of Serbia, Dr. Koste Todorovića 26, 11000 Belgrade, Serbia; 4 Institute of Pathology, School of Medicine, University of Belgrade, Dr. Subotića 1, 11000 Belgrade, Serbia; 5 Clinic for Thoracic Surgery, University Clinical Center of Serbia, Koste Todorovica 10, 11000 Belgrade, Serbia

**Keywords:** non-small cell lung carcinoma, chemotherapy, erlotinib, drug screening, progression-free survival

## Abstract

**Background/Objectives**: Non-small cell lung carcinoma (NSCLC) is characterized by its diverse molecular profiles and varying responses to treatment, highlighting the importance of precision medicine in optimizing therapeutic outcomes. A promising approach involves using patient-derived cellular models, which provide insights into the unique biology of individual tumors and their responsiveness to treatment. **Methods**: We established short-term primary cell cultures from thirteen patients with NSCLC of different subtypes and stages, including both cancer and stromal cells. To evaluate the ex vivo cytotoxicity and selectivity of eight chemotherapeutics and erlotinib, we employed an immunoassay, and the results were analyzed using an automated imaging system. Scoring of the obtained results was also performed. The ex vivo responses to cisplatin, etoposide, and paclitaxel were correlated with the patients’ responses to therapy. We used Kaplan–Meier analysis to assess progression-free survival (PFS) differences among patient groups. **Results**: NSCLC cells exhibited significant variability in their responses to drugs, with stromal cells demonstrating greater sensitivity. Tumors at stages I-III responded to multiple treatments, whereas stage IV cells showed considerable resistance. Erlotinib effectively reduced cancer cell growth at lower doses but plateaued at higher concentrations. The immunoassay indicated 67% sensitivity and 100% specificity in predicting patient responses to chemotherapy. Sensitivity to etoposide and paclitaxel correlated with progression-free survival (PFS). **Conclusions:** A personalized treatment strategy, such as our immunoassay based on the ex vivo responses of cancer patients’ cells, can guide treatment decisions and, in some cases, serve as surrogate biomarkers for tumor types that lack actionable biomarkers.

## 1. Introduction

Lung cancer remains the most common cause of cancer-related mortality worldwide, causing around 1.8 million deaths each year [1]. It is primarily classified into two main categories: non-small cell lung cancer (NSCLC) and small cell lung cancer (SCLC). NSCLC accounts for around 85-90% of all lung cancer cases and is mainly divided into three major subtypes [2]. Adenocarcinoma, which accounts for 40–50% of NSCLC cases, is the most common subtype. It usually develops in the peripheral regions of the lung and is often found in non-smokers. Squamous cell carcinoma represents about 25–30% of NSCLC cases and usually occurs in the central parts of the lung, with a close association with smoking. Large cell carcinoma, which accounts for about 10–15% of NSCLC cases, is characterized by large, abnormal cells. It can occur in any part of the lung and tends to grow and spread rapidly [3]. In addition to the major subtypes, there are several rare subtypes of NSCLC, including pleomorphic carcinoma, giant cell carcinoma, carcinosarcoma, and neuroendocrine carcinoma [4,5]. The accurate classification of NSCLC subtypes is essential for determining appropriate treatment strategies, including targeted therapies and immunotherapy. Advances in targeted therapies and early detection techniques have significantly improved overall survival rates in lung cancer [6,7]. Mortality in NSCLC is falling faster than incidence, as survival rates have improved with the implementation of targeted therapies for specific genomic alterations [5,8]. However, despite advances in diagnostic methods and treatment strategies, the five-year survival rate is still around 10–20%, highlighting the urgent need for new diagnostic and therapeutic approaches [9].

Although advances in chemotherapy have been less dramatic compared to molecular targeted therapy and immunotherapy, chemotherapy remains a cornerstone of lung cancer treatment for all stages, histologic types, mutational subtypes, and immunological statuses [10]. While molecular targeted therapies, such as epidermal growth factor receptor (EGFR) and anaplastic lymphoma kinase (ALK) inhibitors, have significantly helped patients with specific genetic alterations, only a minority of patients with NSCLC have these mutations [11]. In addition, only about 30% of EGFR- and ALK-negative patients have tumors with a PD-L1 tumor proportion of 50% or more, limiting the applicability of immunotherapy for a large proportion of the patient population [12]. Therefore, for the majority of NSCLC patients, chemotherapy remains the primary therapeutic option, the success of which is limited by the fact that individual responses and resistance mechanisms cannot be predicted. Typically, platinum-based therapies such as cisplatin or carboplatin are used in combination with taxanes (paclitaxel or docetaxel), antimetabolites (pemetrexed or gemcitabine), or other agents [13,14,15]. These therapeutics aim to control the tumor and prolong survival, but their efficacy is often compromised by the development of drug resistance and severe side effects. Chemotherapy has also shown clinical benefits in combination with other treatment modalities, including targeted therapies and immunotherapy [16]. The new treatment strategies, such as maintenance therapy and combination therapies, have shown improved survival and reduced toxicity compared with conventional treatments [10,17].

The treatment of NSCLC represents a significant clinical challenge due to the pronounced heterogeneity that can be observed both within individual tumors and among different patients. This complexity underscores the need for personalized treatment strategies that take into account the different molecular and genetic characteristics of each tumor as well as the unique response of a patient’s cancer cells to specific treatments. Traditional methods for determining the best treatment option, such as histopathological analysis and genetic profiling, are limited in their ability to predict individual patients’ tumor responses to therapies. This gap highlights the urgent need for innovative approaches that can provide more precise and tailored treatment options.

In recent years, innovative platforms for studying tumor biology and drug responses have emerged, offering promising solutions for personalized diagnostics and enabling a more nuanced understanding of how specific tumors respond to various therapeutic interventions. Patient-derived organoids (PDOs) and xenografts (PDXs) are widely used preclinical models due to their ability to replicate the tumor microenvironment and retain the genetic heterogeneity of the original tumor [18,19,20]. However, these models are often limited by low throughput, long development times, and technical complexity, making them less suitable for rapid clinical decision making [21]. On the other hand, high-throughput cell-based assays are a viable alternative as they offer the capacity to test a broad spectrum of therapeutic agents under controlled experimental conditions. In this study, we used a novel platform that we previously developed *for* ex vivo drug sensitivity testing with NSCLC patient-derived cells [22]. By correlating patient-specific clinical outcomes, including responses to administered chemotherapy, with the experimental sensitivity of patients’ cancer cells to various chemotherapeutic agents tested on our platform, we provided a comprehensive framework for identifying the most effective treatments. We have shown that this approach has the potential to facilitate the development of personalized chemotherapy regimens by identifying the therapeutic agents that are most likely to benefit individual patients. By integrating clinical data with experimental results, this strategy fills a critical gap in existing diagnostic tools and improves the precision treatment for NSCLC.

## 2. Materials and Methods

### 2.1. Establishment of NSCLC Primary Cell Cultures

Tissue samples from NSCLC patients were obtained from the Thoracic Surgery Clinic at the University Clinical Center of Serbia, with informed consent and ethics committee approval (approval number 623/4). The patients were not treated with medication before surgery. During surgery, tissue samples were examined histopathologically to determine the diagnosis, histological grade, stage, necrosis, and status of lymph node invasion. Histological grades of the NSCLC samples were TR84, TR104, and TR105 (grade I); TR93, TR100, TR159, TR102, and TR106 (grade II); TR64, TR65, and TR153 (grade III); and TR108 and TR109 (grade IV). An EGFR L858R mutation was detected in sample TR64 using the Cobas EGFR Mutation Test v2 (Hoffman-La Roche Ltd., Basel, Switzerland).

The postoperative tissue samples were placed in sterile tubes containing an antibiotic–antimycotic solution (Sigma-Aldrich Chemie GmbH, Taufkirchen, Germany) and taken to the laboratory for processing. The tissue was manually minced and cut into 3–5 mm pieces. It was then enzymatically dissociated using the Tumor Dissociation Kit (Miltenyi Biotec, Bergisch Gladbach, Germany) according to the manufacturer’s protocol. The tissue was incubated in an orbital shaker (KS 4000 ic control, IKA, Königswinter, Germany) at 37 °C and 300 rpm for 90 min.

Dissociated tissues were cultured in DMEM/Ham’s F12 (1:3 ratio) supplemented with 5% fetal bovine serum (Corning, NY, USA), the antibiotic–antimycotic solution, 4 µg/mL hydrocortisone, 1 µg/mL insulin, 10 ng/mL epidermal growth factor (BioLegend, San Diego, CA, USA), and 24 µg/mL adenine (Sigma-Aldrich Chemie GmbH). The growth media were from Sigma-Aldrich Chemie GmbH. Samples were incubated in T-25 cell culture flasks at 37 °C and 5% CO_2_ until cell attachment was observed, followed by medium replacement after attachment.

Successfully established primary NSCLC cultures were maintained for further experiments until confluence, while cultures were discarded if no cell attachment was achieved within 7 days.

### 2.2. Treatments with Chemotherapeutics

Vinorelbine, pemetrexed, and osimertinib were purchased from Selleckchem (Houston, TX, USA). Carboplatin, cisplatin, docetaxel, etoposide, gemcitabine, paclitaxel, and erlotinib were purchased from Sigma-Aldrich Chemie GmbH (Taufkirchen, Germany). Cisplatin, carboplatin, and gemcitabine were prepared in sterile water, while etoposide, docetaxel, vinorelbine, paclitaxel, pemetrexed, erlotinib, and osimertinib were dissolved in DMSO and stored at −20 °C. Before use, all stock solutions were freshly diluted in sterile water. The following concentrations were used for the treatments: vinorelbine (100, 250, 500, 750, and 1000 nM), pemetrexed (50, 75, 100, 200, and 300 µM), carboplatin (10, 25, 50, 75, and 100 µM), cisplatin (5, 7.5, 10, 12.5, and 15 µM), docetaxel (1, 2, 3, 4, and 5 µM), etoposide (10, 15, 20, 25, and 30 µM), gemcitabine (10, 25, 50, 75, and 100 µM), paclitaxel (1, 2, 3, 4, and 5 µM), erlotinib (0.5, 1, 2, 3, and 4 µM), and osimertinib (50, 75, 100, 125, and 150 nM). Primary cells were plated in black clear bottom, 384-well cell culture microplates (Thermo Fisher Scientific, Waltham, MA, USA) in 50 µL of the cell growth medium at a density of 1000 cells per well. Treatments were initiated 72 h after seeding and lasted for 7 days.

### 2.3. Immunofluorescence Assay

The immunofluorescence assay was fine-tuned to differentiate cancer cells from stromal cells by employing a combination of CK8/18 antibodies. Additionally, the cells were co-stained using a mouse antibody targeting ABCB1 [22].

Cells were fixed for 20 min at room temperature in 4% paraformaldehyde and subsequently washed with the Well-wash™ Versa microplate washer (Thermo Fisher Scientific, Waltham, MA, USA). Following fixation, cells were treated with a blocking solution containing 2% bovine serum albumin (BSA) in PBS for one hour at room temperature. Next, cells were incubated overnight at 4 °C with a primary antibody mixture comprising a rabbit CK8/18 antibody cocktail (clone SU0338, #MA5-32118, Thermo Fisher Scientific, Waltham, MA, USA) and a primary mouse antibody against ABCB1 (clone C219, #MA1-26528, Thermo Fisher Scientific, Waltham, MA, USA). Afterwards, cells were exposed at room temperature for two hours to the following secondary antibodies: Alexa Fluor 555 goat anti-mouse (#A-21422, Thermo Fisher Scientific, Waltham, MA, USA) and Alexa Fluor 488 goat anti-rabbit (#A-11008, Thermo Fisher Scientific, Waltham, MA, USA), under conditions that protect the cells from light. For nuclear visualization, cells were counterstained with 1 µg/mL Hoechst 33342 for two hours at room temperature. Prior to imaging, cells were kept at 4 °C in the dark.

Fluorescently labeled cells were imaged using the ImageXpress^®^ Pico Automated Cell Imaging System (Molecular Devices^®^, San Jose, CA, USA) equipped with a 4x objective. Image analysis was carried out using CellReporterXpress^®^ software version 2.8.2.669 (Molecular Devices^®^). To evaluate the cytotoxic effects of the drug, the cell scoring analysis protocol was implemented, while the expression of the ABCB1 transporter was assessed using the multi-wavelength cell scoring analysis protocol described in the previous study [22].

**Scheme 1:**
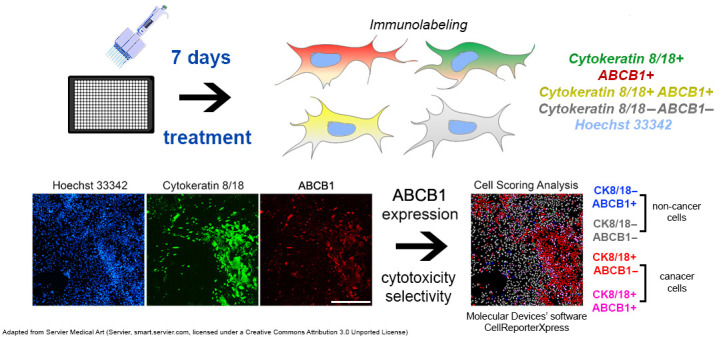
Assessment of cytotoxicity, selectivity towards cancer cells, and ABCB1 expression in patient-derived NSCLC cells. Scale bar = 700 µm.

To precisely segment nuclear Hoechst 33342 and cytoplasmic CK8/18 staining, we established the minimum and maximum widths for both the nucleus and the entire cell, along with the appropriate signal intensity thresholds. The analysis provided counts for total cells (Hoechst 33342-positive), cancer cells (Hoechst 33342-positive and CK8/18-positive), and non-cancer cells (Hoechst 33342-positive and CK8/18-negative), as well as for the cancer and non-cancer cells that are either positive or negative for ABCB1 expression in each well of the microplate (Scheme 1). We statistically evaluated the immunofluorescence assay data (counts of four distinct groups of cells as indicated in Scheme 1) using GraphPad Prism software version 8.0.2. The IC50 value, representing the drug concentration needed to reduce cell growth by 50%, was determined through non-linear regression analysis on the entire population of cancer cells and the entire population of non-cancer cells using the log (inhibitor) versus normalized response model within GraphPad Prism 8.0.2.

The response of NSCLC patients’ cells to treatment was assessed using a modified scoring system described in our previous article [23].

An overall drug response score was calculated by integrating various weighted parameters. This score was determined by comparing the effects of drugs on cancer cells and stromal cells via an immunoassay with the following criteria:

1. Inhibitory Effect at Maximum Dose: The percentage inhibition at the highest dose is multiplied by 0.1. If stromal cells show a greater inhibitory effect, the value is multiplied by −0.1.
2. Dose for 10% Inhibition (IC10): This dose is multiplied by 0.05. If the dose required for cancer cells is higher than that for stromal cells, it is multiplied by −0.05.
3. Dose for 25% Inhibition (IC25): this dose is also multiplied by 0.05, with the same adjustment made if the cancer cells require a higher dose than the stromal cells.
4. Dose for 50% Inhibition (IC50): The dose is multiplied by 0.1 unless it exceeds 100 µM, in which case the factor is set to 0. If stromal cells require a lower dose, the value is multiplied by −0.1 or by −10 if the dose exceeds 100 µM.
5. Dose for 75% Inhibition (IC75): The dose is multiplied by 0.05 and reduced to 0 if it exceeds 100 µM. If stromal cells require a lower dose, the value is adjusted to −0.05 or −5 if the dose exceeds 100 µM.
6. Dose for 90% Inhibition (IC90): similar to IC75, the dose is multiplied by 0.05, adjusted to 0 above 100 µM, and set to −0.05 or −5 if stromal cells require a lower dose.
7. Slope
8. Area Under the Curve (AUC): this value is multiplied by 0.35.
9. Stimulation of Cancer Cell Growth: If none of the tested doses stimulates cancer cell growth, the lowest dose is multiplied by 0.05. If growth stimulation is observed (up to 125%), the same factor is applied. Growth stimulation between 125% and 150% is multiplied by 0, and stimulation above 150% is multiplied by −0.05.
10. Breakthrough Dose: If the drug has a rapid effect at lower doses but only a limited effect at higher doses, the breakthrough dose is multiplied by −0.05. Conversely, in the case of a dose-dependent effect, the dose that causes 50% inhibition is multiplied by 0.05.
11. Incomplete Inhibition at Maximum Dose: If more than 25% of the cancer cells remain viable at the highest dose, the value is multiplied by −0.05. For a viability of 10% to 25% the multiplier is 0, and for less than 10%, the multiplier is 0.05.

All individually weighted parameters were summed to calculate the “drug response score”. A score between 0 and 40 indicates that the treatment is more effective against cancer cells, while a score between 0 and −40 indicates a stronger effect on stromal cells. The R package PharmacoGx was used to calculate the dose–response metrics, including the slope and area under the curve (AUC). The compute AUC function from this package was used specifically to determine the AUC for the drug dose–viability curve. This function takes drug concentration and cell viability as inputs, normalizing the AUC based on the concentration range. The AUC, which reflects the response area (1—Viability), is calculated as the area under the curve on a log10 concentration scale. A higher AUC value indicates an increased sensitivity to the drug and is therefore a crucial parameter for evaluating the efficacy of the treatment.

### 2.4. Barnard’s Exact Test

A two-sided Barnard’s exact test, which offers more statistical power for small sample sizes than Fisher’s exact test, was utilized to analyze the correlation between ex vivo responses and patients’ responses to adjuvant chemotherapy. Patients were divided into two groups: those who were sensitive and did not relapse within the first 12 months of follow-up and those who were resistant and experienced a relapse within the same period.

### 2.5. Progression-Free Survival via Kaplan–Meier Analysis

The Kaplan–Meier method was used to evaluate the statistical differences in progression-free survival (PFS) among various patient groups. These groups included those sensitive vs. resistant to the tested drugs, those with or without lymph node invasion, patients at stages I and II vs. those at stages III and IV, and adenocarcinoma subtypes compared to other NSCLC subtypes. Data were collected regarding when the patients entered the study, the timing of events (progression of the disease), and when the study was completed. Survival probabilities were calculated based on the number of events, which included four patients exhibiting PFS at months 3, 6, and 12. There were also nine patients whose progression-free survival data were censored after 12, 15, 18, and 22 months of follow-up.

## 3. Results

### 3.1. Clinical Parameters of NSCLC Patients

Table 1 provides a summary of 13 NSCLC patients enrolled in this study. The tissue samples from patients were used to establish primary cultures. The patients included ten men and three women. Their age ranged from 51 to 74 years. Neither neoadjuvant chemotherapy nor radiotherapy has been administered. The pathological stages encompassed three patients with stage I, five patients with stage II, three patients with stage III, and two patients with stage IV. Tumors included eight adenocarcinomas, four squamous cell carcinomas, and one large cell neuroendocrine carcinoma. In the TR64 patient, an EGFR L858R mutation was detected. After surgical resection, the majority of patients received three cycles of cisplatin–etoposide chemotherapy. In addition to cisplatin–etoposide chemotherapy, patient TR64 received osimertinib. Patients with stage IV, namely TR108 and 109, received five and six cycles of cisplatin–paclitaxel chemotherapy, respectively. After a relapse, TR108 was treated with cisplatin and gemcitabine. After two cycles, the patient experienced chemotoxicity and subsequently proceeded to atezolizumab. Before receiving chemotherapy, patient TR159 experienced disease progression and proceed with pebrolizumab therapy.

### 3.2. Ex Vivo Evaluation of the Sensitivity of Patient-Derived Cells to Chemotherapy and Erlotinib

**Figure 1:**
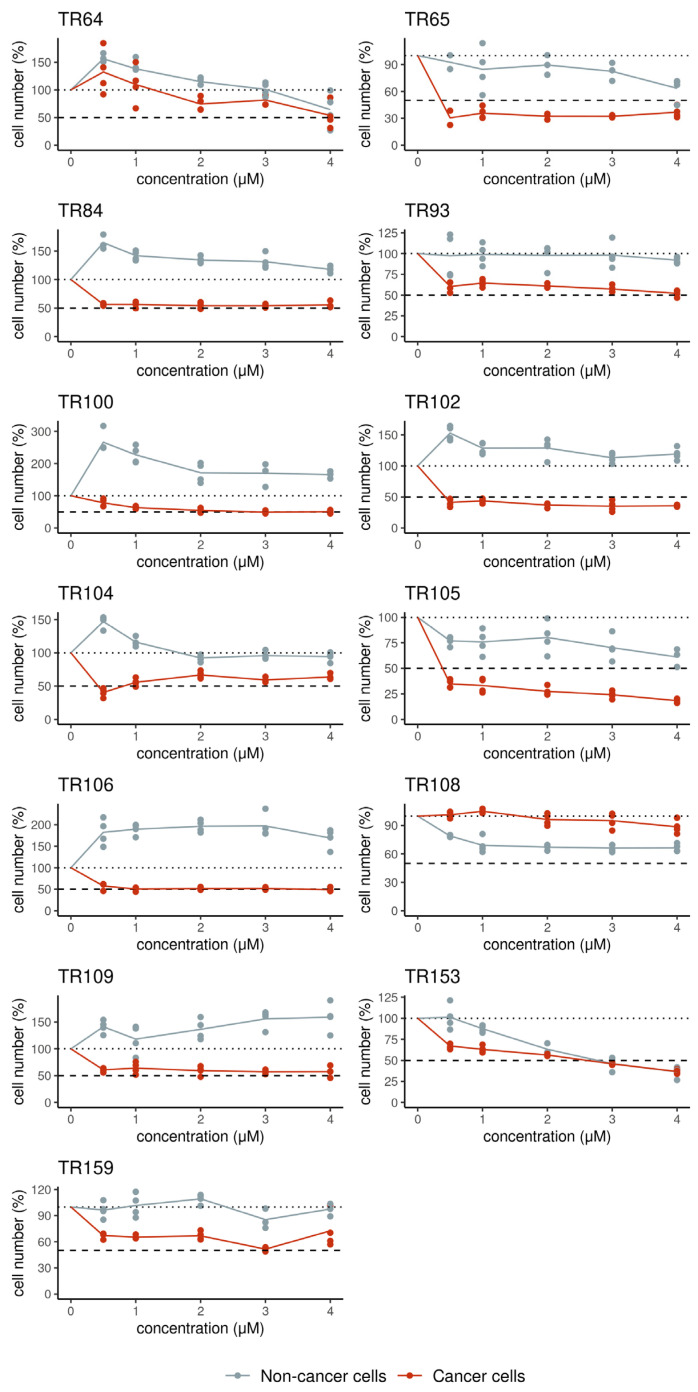
Erlotinib’s selectivity towards cancer cells. The cytotoxic effects of erlotinib were assessed in cultures derived from 13 patients with NSCLC. The CK8/18 antibody was utilized to differentiate between cancer and stromal (non-cancer) cells in a mixed culture. The treatment with erlotinib was conducted over a period of 7 days. Data are presented as the mean ± SEM, with n = 4 for each measurement. Dashed lines indicate 50% and 100% of viable cells, emphasizing the cytotoxicity and selectivity of erlotinib towards cancer cells.

The patient-derived NSCLC cells were treated with cisplatin, carboplatin, etoposide, pemetrexed, gemcitabine, paclitaxel, docetaxel, and vinorelbine. The IC50 values for each drug are shown in Table 2. The patient-derived cells showed variability in their responses to chemotherapeutics, demonstrating different drug sensitivities and resistance patterns. Among the examined chemotherapeutics, only pemetrexed was generally ineffective against cancer cells in NSCLC cultures. While some cultures displayed selective cytotoxicity of applied chemotherapeutics towards CK8/18+ cancer cells, in most cultures, CK8/18− non-cancer cells were more sensitive to chemotherapeutics (Table 2), suggesting a potential for increased cytotoxic effects on normal tissues. Cancer cells from patients with stage I (TR84 and TR105), stage II (TR100, TR106 and TR159), and stage III (TR64 and TR153) were sensitive to applied chemotherapeutics in a tested range of concentrations (Table 2). However, cancer cells from stage IV patients (TR108 and TR109) were resistant to most tested chemotherapeutics (Table 2). The patient-derived NSCLC cultures were also treated with the EGFR inhibitor erlotinib. As shown in Figure 1, erlotinib exhibited notable efficacy across all NSCLC samples. Erlotinib selectively inhibited the growth of cancer cells in NSCLC cultures compared to stromal cells. In most patient samples, erlotinib achieved 50% inhibition of cancer cell proliferation at lower concentrations. However, its cytotoxicity did not increase with increased concentrations, demonstrating what is known as a plateau effect (Figure 1).

### 3.3. Scoring of Patients’ Cell Responses to Chemotherapeutics and Erlotinib

**Figure 2:**
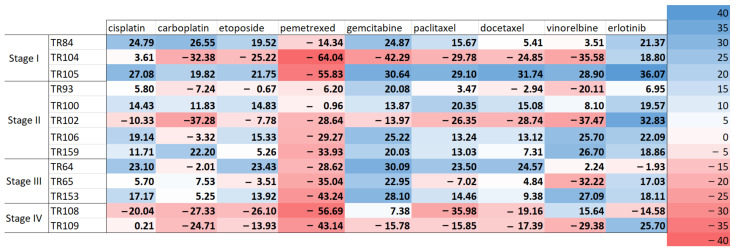
NSCLC patient-derived cell cultures’ sensitivity is illustrated by the scoring of the drugs’ efficacy. A drug sensitivity assessment was conducted for 13 NSCLC cultures derived from patients by testing their response to 8 different chemotherapeutic agents and erlotinib. The scoring system ranges from 0 to 40, where higher scores represent more significant toxicity to cancer cells. Conversely, scores between 0 and −40 indicate situations where stromal (non-cancer) cells exhibit higher sensitivity than cancer cells. Scores above 10 are regarded as favorable responses.

Through the comparison of drug effects on cancer and stromal cells obtained by immunoassay, the drug response scores of NSCLC patients’ samples were generated using a modified scoring method. The scoring results show the most beneficial drugs for each patient based on their cytotoxicity and selectivity (Figure 2). A score equal to or above 10 indicates the sensitivity of a patient’s sample to a specific drug. A score below 10 signifies resistance to that drug. Among the cohort of NSCLC patients’ samples, the best response was observed in TR105 (sensitivity towards erlotinib and seven chemotherapeutics). Patients’ samples with stage I (TR84) and stage II (TR100) also showed good responses to five and six chemotherapeutics, respectively, and erlotinib. Other patients’ samples exhibited variable responses to the applied drugs. However, samples with stage IV did not show sensitivity to most drugs. TR108 responded favorably only to vinorelbine, while TR109 responded well to erlotinib. The scoring indicated that erlotinib was the most beneficial drug for the patients examined, regardless of the NSCLC subtype, disease stage, or EGFR mutation status, with 10 responders (Figure 2). Interestingly, erlotinib was not effective for the patient sample TR64 with the EGFR mutation (Table 1, Figure 2). However, this patient had a progression-free survival (PFS) of more than 22 months and was treated with osimertinib (Table 1). The second most beneficial drug was gemcitabine, with nine responders. Both cisplatin and paclitaxel had seven responders each, followed by etoposide with six responders and vinorelbine with five responders. Carboplatin and docetaxel each had four responders, while none of the tested samples responded to pemetrexed (Figure 2).

### 3.4. Correlation of Drug Response Scores of Patients’ Samples with Patients’ Responses to Therapy

Based on the scoring results from Figure 2 and considering that NSCLC patients received adjuvant chemotherapy with cisplatin–etoposide or cisplatin–paclitaxel (only considered for TR108 and TR109), we categorized the ex vivo responses to these drugs as either sensitive or resistant, as outlined in Table 3. We defined sensitivity to therapy as occurring when a relapse occurred more than 12 months after surgery. Conversely, if a relapse occurred at 12 months or earlier, the patients were classified as resistant to treatment (Table 3). We categorized patient samples based on their inherent ABCB1 expression, identifying those with at least 20% expression as resistant (Table 3).

We noted an overlap with the ex vivo-sensitive samples; all six sensitive samples were associated with patients who did not experience disease progression within 12 months, indicating a positive response to the adjuvant chemotherapy (Table 3). Regarding the ex vivo-resistant samples, four patients showed disease progression at 12 months or earlier. However, we could not establish a consistent relationship with three ex vivo-resistant samples, as these patients responded well to the adjuvant chemotherapy. Two patients with inherently expressed ABCB1 transporter were resistant to chemotherapy and experienced disease progression. According to the expression of ABCB1, sensitive profiles were more aligned with the patient’s outcomes.

Barnard’s exact test of our small-size sample cohort revealed a significant correlation between ex vivo responses and the responses of NSCLC patients to adjuvant chemotherapy (Table 4). However, the sensitivity of our immunoassay indicates a 67% (six out of nine, Table 4) positive prediction rate for identifying responders among NSCLC patients. This means that when the ex vivo test shows a response, there is a 67% chance that the patient is genuinely a responder. The specificity stands at 100% (four out of four, Table 4), confirming that patients’ samples identified as non-responders indeed do not exhibit a response in the real world. Overall, our assay has an accuracy of 77%, demonstrating better performance in detecting non-responders.

### 3.5. Progression-Free Survival Is Associated with Ex Vivo Sensitivity to Chemotherapeutics

**Figure 3:**
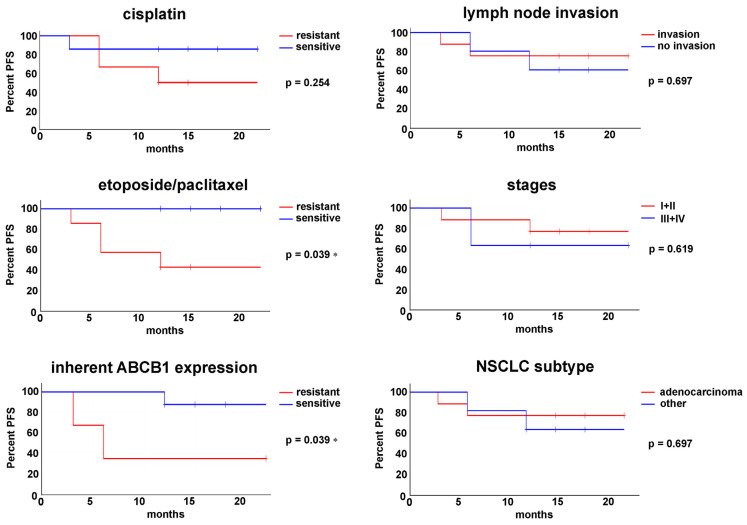
Progression-free survival (PFS) differences among various groups of patients. Kaplan–Meier survival curves illustrate the PFS among different patient groups categorized by drug sensitivity (cisplatin and etoposide/paclitaxel), inherent ABCB1 expression, lymph node invasion status, cancer stages (I + II vs. III + IV), and NSCLC subtypes (adenocarcinoma vs. other). Corresponding *p* values are also presented. Statistically significant difference in PFS among different patient groups are indicated by * (*p* < 0.05).

Using Kaplan–Meier analysis (Figure 3), we demonstrated a statistically significant difference in progression-free survival (PFS) when comparing sensitive patient samples versus resistant to etoposide/paclitaxel treatment, and according to the ABCB1 expression as outlined in Table 3. While a difference was also observed between sensitive and resistant samples to cisplatin, this finding did not reach statistical significance. Factors such as lymph node invasion, NSCLC staging, and NSCLC subtype classification did not significantly influence PFS outcomes (Figure 3).

## 4. Discussion

Despite the advancements made in precision medicine, numerous challenges continue to persist. Cancer patients harboring druggable genetic targets may exhibit variable responses to personalized treatment regimens. This variability complicates associating a tumor’s molecular profile with corresponding effective therapies.

Lung cancer, characterized by significant genetic heterogeneity, presents considerable challenges for implementing precision medicine [24]. Currently, personalized treatment regimens rely heavily on molecular analyses, mainly via next-generation sequencing technologies [25]. NSCLC remains the most extensively studied lung cancer; however, only approximately 30% of patients possess druggable mutations, and not all individuals with these mutations exhibit a beneficial response to targeted therapies [26]. Specifically, a subtype of NSCLC, namely lung squamous cell carcinoma, lacks identifiable biomarkers and does not express any druggable oncogenic drivers. Notably, some patients devoid of druggable mutations have been found to respond positively to agents such as EGFR tyrosine kinase inhibitors [23,27].

Establishing preclinical models that accurately reflect the characteristics of the original tumor is imperative to effectively predict clinical outcomes associated with targeted therapies. Recent investigations into patient-derived cancer models underscore their reliability and potential utility as a scalable platform for preclinical testing [28].

This study explored the efficacy of various chemotherapeutics and erlotinib using patient-derived cell cultures from NSCLC patients, including cancer and stromal cells. Utilizing a specialized immunoassay developed in our laboratory [22], we aimed to establish a correlation between ex vivo drug responses and actual patient outcomes.

Our findings reveal significant variability in how different patient-derived NSCLC cells respond to chemotherapeutics. We observed that stromal cells exhibited increased sensitivity to many of the drugs tested, suggesting that these treatments might harm normal tissues and raising concerns regarding their therapeutic index. The sensitivity of NSCLC cells to the selected drugs varied across disease stages; stages I through III demonstrated a range of responses, while stage IV cells displayed considerable resistance, underscoring the challenges of treating advanced diseases.

Among the drugs tested, the EGFR inhibitor erlotinib was effective across all NSCLC samples. It selectively reduced cancer cell proliferation while sparing the surrounding stromal cells, showcasing substantial inhibition at lower doses. However, the lack of enhanced cytotoxic effects at higher concentrations suggested a plateau effect, indicating a potential limitation in fully harnessing its therapeutic benefits. This finding highlights the need for dosage optimization and the exploration of combination therapies, particularly for patients with resistant NSCLC.

Our immunoassay results indicated distinct drug response profiles based on the disease stage. Specifically, stage I (TR84) and stage II (TR100) samples showed positive responses to multiple chemotherapeutics, including erlotinib, emphasizing their effectiveness in earlier disease stages. Conversely, stage IV samples exhibited notable drug resistance, with only one specific agent eliciting a positive response. Erlotinib emerged as the most beneficial option for all patients, irrespective of the NSCLC subtype or EGFR mutation status. Gemcitabine was the second most effective drug. Other drugs, such as cisplatin and paclitaxel, demonstrated substantial efficacy, while pemetrexed showed no responses, raising questions about its suitability for NSCLC patients.

Our research revealed a significant correlation between ex vivo drug responses and the clinical effectiveness of adjuvant chemotherapy in NSCLC patients. The immunoassay exhibited a sensitivity of 67% (a positive prediction rate for identifying responders), suggesting that a favorable ex vivo test corresponds with a genuine therapeutic response in two-thirds of cases. The assay’s 100% specificity further confirmed that all non-responders were accurately predicted, contributing to an overall accuracy rate of 77%. This emphasizes its potential as a tool for enhancing treatment strategies for NSCLC patients.

We observed a noteworthy distinction in progression-free survival (PFS) among patient samples classified as sensitive vs. resistant to etoposide/paclitaxel treatment, suggesting that sensitivity to etoposide/paclitaxel could serve as a surrogate biomarker for predicting patient outcomes.

Our investigation found that factors like lymph node invasion, NSCLC staging, and subtype classification did not significantly impact PFS outcomes. However, the level of ABCB1 transporter expression did result in significant differences in PFS outcomes. This raises questions about the influence of tumor biology and resistance markers such as ABCB1 on treatment responses, suggesting that these variables may require further exploration in a larger patient cohort. Overall, these findings support a personalized approach to treatment based on individual tumor characteristics and ex vivo responses.

One of the primary limitations of our study was the heterogeneity of the patient cohort. As a real-world investigation, the absence of a structured enrollment strategy resulted in variability in participants’ genomic backgrounds and treatment protocols. The limited sample size for drug sensitivity assessments and inconsistencies in treatment regimens constrained some of our findings. Additionally, we did not investigate the combined effect of two chemotherapeutics, even though combination regimens were a part of the standard care for some patients. This study is part of a larger project aimed at screening a broad range of chemotherapeutics and tyrosine kinase inhibitors, reflecting our commitment to innovative treatment strategies and improving patient outcomes.

In contrast to many studies that emphasize the long-term cultivation of patient-derived lung cancer models or the establishment of living biobanks, our research concentrated on the rapid evaluation of drug responses as a translational medicine strategy. Consequently, all primary cell cultures were established and tested within a short timeframe of two to three weeks from patient sampling.

Overall, this study underscores the practical application of ex vivo drug screening in tailoring precision therapy for NSCLC and lays the groundwork for future clinical trials aiming to enhance treatment efficacy and patient outcomes.

## 5. Conclusions

This study highlights the complexity and variability in drug responses among NSCLC patients, emphasizing the challenges encountered in precision medicine. The findings reveal that while erlotinib shows potential across a range of NSCLC samples, including those lacking identifiable druggable mutations, the overall response to chemotherapy is influenced by multiple factors, such as the stage of the disease and the sensitivity of stromal cells.

The positive correlation between ex vivo drug responses and clinical effectiveness suggests that our specialized immunoassay could be a valuable tool in optimizing treatment strategies, thereby enhancing the predictability of therapeutic responses.

The efficacy of specific agents, particularly erlotinib and gemcitabine, during earlier disease stages underscores the significance of early intervention in managing NSCLC. The notable differences in progression-free survival among patients based on their sensitivity to chemotherapeutics imply that these sensitivities may serve as surrogate biomarkers, facilitating better-informed treatment decisions.

## Figures and Tables

**Table 1:** Clinical parameters of NSCLC patients included in our study.

Patient	Sex	Age	NSCLC Subtype	Lymph Node Invasion	Stage	EGFR Status	Chemotherapy	Chemotherapy After Relapse
**TR64**	male	71	adenocarcinoma	N2	IIIA	Mutation L858R	cisplatin + etoposide, 3 cycles, osimertinib	/
**TR65**	male	51	large cell neuroendocrine carcinoma	N0	IIIA	wt	cisplatin + etoposide, 3 cycles	cisplatin + etoposide, 2 cycles
**TR84**	female	58	squamous cell carcinoma	N0	IB	wt	cisplatin + etoposide, 3 cycles	/
**TR93**	male	72	squamous cell carcinoma	N0	IIA	wt	cisplatin + etoposide, 3 cycles	/
**TR100**	male	61	adenocarcinoma	N0	IIA	wt	cisplatin + etoposide, 3 cycles	/
**TR102**	male	55	adenocarcinoma	N0	IIB	wt	cisplatin + etoposide, 3 cycles	/
**TR104**	male	68	adenocarcinoma	N0	IB	wt	cisplatin + etoposide, 2 cycles	/
**TR105**	male	66	squamous cell carcinoma	N0	IB	wt	cisplatin + etoposide, 3 cycles	/
**TR106**	female	74	squamous cell carcinoma	N0	IIB	wt	cisplatin + etoposide, 3 cycles	/
**TR108**	male	65	adenocarcinoma	N0	IV	wt	cisplatin + paclitaxel, 5 cycles	cisplatin + gemcitabin, 2 cycles—chemotoxicity, proceed with atezolizumab
**TR109**	male	59	adenocarcinoma	N1	IV	wt	cisplatin + paclitaxel, 6 cycles	/
**TR153**	female	68	adenocarcinoma	N2	IIIA	wt	cisplatin + etoposide, 3 cycles	/
**TR159**	male	63	adenocarcinoma	N1	IIA	wt	/	pembrolizumab, 5 cycles

**Table 2:** IC_50_ values of chemotherapeutics in NSCLC patient-derived cultures.

NSCLC Cultures		IC50 µM									
**Cancer Cells** **(CK8/18+)**		**Cisplatin**	**Carboplatin**			**Etoposide**	**Pemetrexed**	**Gemcitabine**	**Paclitaxel**	**Docetaxel**	**Vinorelbine**
**Stage I**	TR84	2.11 *	8.15 *			2.98 *	>300 *	2.68 *	2.57 *	1.85 *	0.18 *
	TR104	12.24	>100			>30	>300	>100	>5	>5	>1
	TR105	2.12	7.51			4.37	>300	2.77	0.49	0.49	0.05
**Stage II**	TR93	9.02	55.27			20.94	>300	7.37	2.63	3.71	0.65
	TR100	3.87	15.57			5.46	54.79	7.61	0.51	0.54	0.16
	TR102	16.30	>100			>30	>300	44.37	>5	>5	>1
	TR106	4.19	38.64			13.59	>300	2.68	2.69	2.76	0.33
	TR159	6.22	20.60			11.98	>300	9.79	1.57	1.58	0.34
**Stage III**	TR64	6.07 *	38.98 *			11.92 *	251.86 *	4.92 *	1.35 *	1.56 *	0.17 *
	TR65	14.64 *	22.49 *			25.39 *	231.47 *	4.91 *	1.18 *	>5 *	>1 *
	TR153	4.95	30.88			7.35	>300	5.24	1.31	1.33	0.17
**Stage IV**	TR108	>15	>100			>30	>300	11.01	>5	>5	>1
	TR109	14.85	>100			>30	>300	63.96	>5	>5	>1
**Non-Cancer Cells (CK8/18−)**		**Cisplatin**	**Carboplatin**			**Etoposide**	**Pemetrexed**	**Gemcitabine**	**Paclitaxel**	**Docetaxel**	**Vinorelbine**
**Stage I**	TR84	0.79 *	1.28 *			1.01 *	>300 *	0.49 *	0.31 *	0.18 *	0.03 *
	TR104	6.42	48.56			15.61	>300	25.80	2.71	2.71	0.25
	TR105	1.35	6.32			2.95	185.80	0.73	1.54 ^S^	1.04 ^S^	0.28 ^S^
**Stage II**	TR93	4.29	21.01			7.06	>300 ^S^	0.82	2.03	1.11	0.28
	TR100	1.88	3.40			1.67	44.86	1.48	0.07	0.06	0.04
	TR102	5.18	38.40			12.30	>300	7.50	1.76	2.27	0.13
	TR106	2.23	15.20			7.11	>300	0.46	3.22 ^S^	2.27	0.53 ^S^
	TR159	0.73	17.31			1.58	>300	1.90	0.29	0.19	048 ^S^
**Stage III**	TR64	8.26 *^S^	12.76 *			18.13 *^S^	>300 *^S^	7.06 *^S^	1.95 *^S^	2.73 *^S^	0.18 *
	TR65	1.21 *	2.16 *			2.08 *	25.18 *	0.35 *	1.40 *	0.46 *	0.03 *
	TR153	1.04	4.98			1.26	>300	0.82	1.00	0.66	0.52 ^S^
**Stage IV**	TR108	11.47	90.12	>30	>300	6.07	>5	>5	>1 ^S^		
	TR109	6.49	38.14	12.28	>300	9.22	3.72	2.62	0.19		

* [22], ^S^ selectivity towards cancer cells.

**Table 3:** Ex vivo responses in association with NSCLC patients’ responses to adjuvant chemotherapy.

Patient	Categorization of Ex Vivo Responses to Cisplatin *	Categorization of Ex Vivo Responses to Etoposide/Paclitaxel *	Categorization According to the Inherent Expression of the ABCB1 Transporter #	Progression-Free Survival (PFS)
**TR64**	sensitive	sensitive	resistant	>22 months
**TR65**	resistant	resistant	resistant	6 months
**TR84**	sensitive	sensitive	sensitive	>18 months
**TR93**	resistant	resistant	sensitive	12 months
**TR100**	sensitive	sensitive	sensitive	>18 months
**TR102**	resistant	resistant	sensitive	>15 months
**TR104**	resistant	resistant	sensitive	>15 months
**TR105**	sensitive	sensitive	sensitive	>15 months
**TR106**	sensitive	sensitive	sensitive	>12 months
**TR108**	resistant	resistant	no data	6 months
**TR109**	resistant	resistant	sensitive	>12 months
**TR153**	sensitive	sensitive	no data	>12 months
**TR159**	sensitive	resistant	resistant	3 months

* A score of 10 or higher indicates that a patient’s sample is sensitive to a particular drug, while a score below 10 signifies resistance to that drug, as indicated in Figure 2. # Patient samples with at least 20% ABCB1 expression are considered as resistant.

**Table 4:** Barnard’s exact test of correlation between ex vivo responses and patients’ responses to adjuvant chemotherapy.

*p* = 0.031006 #	Ex Vivo Sensitive	Ex Vivo Resistant	Total
**Clinically** ** sensitive** **(relapse after 12 months from surgery)**	6	3	9
**Clinically resistant** **(relapse within 12 months from surgery)**	0	4 #	4
**Total**	6	7	13

# Since TR159 was sensitive to cisplatin but resistant to etoposide/paclitaxel, we also considered it as a resistant sample.

## Data Availability

The data presented in this study are available upon reasonable request to the corresponding author.

## Abbreviations

The following abbreviations are used in this manuscript:

ABCB1	ATP-binding cassette transporter B1
ALK	Anaplastic lymphoma kinase
BSA	Bovine serum albumin
CK8/18	Cytokeratin (8/18)
DMEM	Dulbecco’s Modified Eagle Medium
DMSO	Dimethyl sulfoxide
EGFR	Epidermal growth factor receptor
IC50	Half-maximal inhibitory concentration
NSCLC	Non-small cell lung carcinoma
PBS	Phosphate-buffered saline
PDXs	Patient-derived xenografts
PDOs	Patient-derived organoids
PD-L1	Programmed death-ligand 1
PFS	Progression-free survival
SCLC	Small cell lung carcinoma
SEM	The standard error of the mean
